# Supplementary figures and images for: Analysis of KSHV B lymphocyte lineage tropism in human tonsil reveals efficient infection of CD138+ plasma cells
Source: PLoS Pathog. 2020 Oct 19;16(10):e1008968. doi: 10.1371/journal.ppat.1008968 (PMC7595638; doi:10.1371/journal.ppat.1008968)

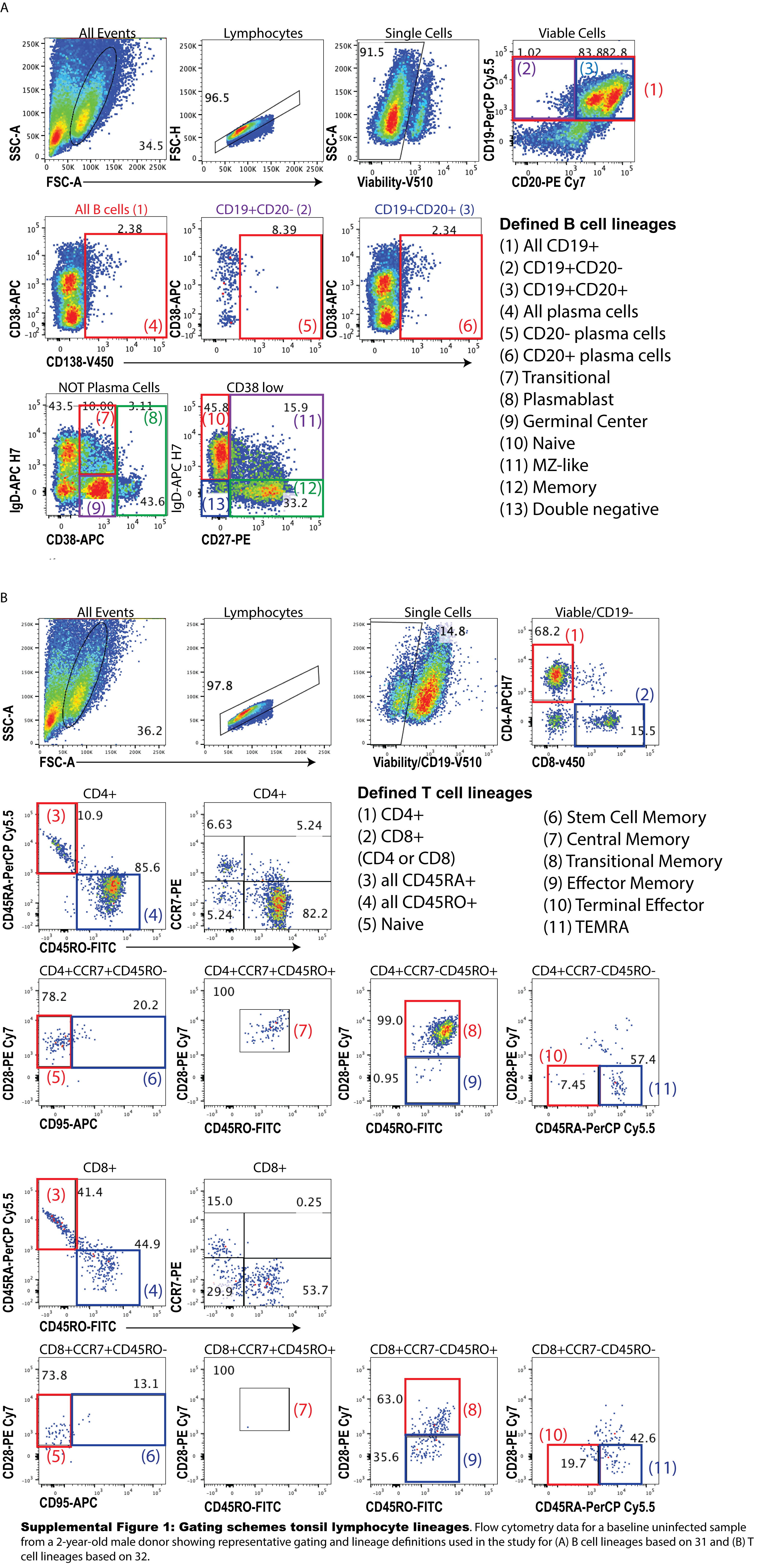

Supplement: S1 Fig — Flow cytometry data for a baseline uninfected sample from a 2-year-old male donor showing representative gating and lineage definitions used in the study for (A) B cell lineages based on vanZelm et. al. 2007 [43] and (B) T cell lineages based on Mahnke et. al. 2013 [44]. (TIF) [file ppat.1008968.s001.tif]
