# Supplementary material for: Analysis of KSHV B lymphocyte lineage tropism in human tonsil reveals efficient infection of CD138+ plasma cells
Source: PLoS Pathog. 2020 Oct 19;16(10):e1008968. doi: 10.1371/journal.ppat.1008968 (PMC7595638; doi:10.1371/journal.ppat.1008968)
Supplement: Attachment — Submitted filename: D-20-00534_Response.docx [file ppat.1008968.s004.docx]

PPATHOGENS-D-20-00534 Response to Reviewers

We would like to thank the reviewers for their thorough reading of our study and their insightful comments which gave us an opportunity to improve our manuscript. In response to these comments we have substantially revised the manuscript and have included a significant amount of additional data and analysis to the study. We believe that the manuscript has been substantially improved by these revisions.

This document contains the original reviewer comments (indicated throughout by italic font and light grey shading), followed by our response to the comment (non-italic, non-shaded font) and a description of any actions we took to revise the manuscript in response to the comment.

**Part 1: Summary**

*Reviewer #1: The authors have analyzed KSHV infection of 40 human tonsils, focusing on infection of tonsillar B cells. Their approach was methodical (though did involve freezing and thawing the lymphocytes prior to infection) and employed a feeder cell system to maintain lymphocyte viability that they had published previously. Their main finding was that KSHV appears to have a strong predilection for CD138+ cells, which is of interest since one of the main diseases from KSHV (MCD) are exclusively CD138+. The strength of this work is the careful flow-cytometry based analysis of multiple B cell subtypes in coming to this conclusion. The other cell types really did not compare in terms of susceptibility to infection.*

*Their approach (negative selection of B cells) involved the post-infection addition of cells originally selected against. This is artificial since it is a far cry from the natural T/B/connective tissue environment of the tonsil's microenvironment but, nevertheless, did enable their detailed B cell subtype analyses.*

*In addition, the investigators examined the role of CD138 as an actual viral receptor, critical to the tropism that they noted. Their results were inconclusive and they concluded that other factors likely play a role.*

*One potential weakness in their overall system is their dependence on a red/green reporter system and a single (3 dpi) timepoint that would allow GFP signal persistence. See "Major issues" below. The authors do not, at least in this study on B cell subtypes, verify that their GFP expression represents true ("stable") infection with viral gene expression. The KSHV.219 GFP is a marvelous surrogate marker for entry but reflects transcription of a cellular promoter. Further, the authors criticized other studies in the Discussion for looking at only latent or lytic gene expresso but then only analyzed GFP, which represents neither. (The authors could have examined their cells for lytic activity easily by looking for red fluorescence and may have but didn't report it .) Finally, if lytic activity did occur, CD138 by 3dpi could be down-regulated below flow cytometric detection infection, as others have shown with reactivation systems. Though unlikely, such down-regulation could possibly skew their conclusion.*

We agree with the reviewer that the GFP reporter isn’t the ideal output for stable infection, however, the technical limitations of the primary lymphocyte system make it a necessary and useful surrogate for the purposes of our multi-color flow cytometry system. In our previous PLoS Pathogens publication, (Totonchy et. al. 2018, Figure 1C) we demonstrated the presence of viral transcripts at 2, 4, and 6 days post-infection with both lytic and latent transcripts appearing at similar levels in the same tonsil. These results established that GFP is a reliable marker for true infection and not only representative of abortive entry events. Moreover, these previous results demonstrate that that there is a mixture of both lytic and latent infection occurring in each tonsil. In this study, we restricted our analysis to 3dpi in order to observe the establishment of infection in various B cell lineages while minimizing the potential for virus-mediated alterations in cellular immunophenotypes (which we observed in our previous study) creating issues with the lineage analysis. We have added text to the results section (lines 105-108) to clarify the choice of timepoint.

In response to the reviewer’s concern that GFP positive cells do not constitute true infection in our current study, we have added data to the manuscript showing qPCR analysis of total RNA extracted from 20 of the 40 tonsil samples included in the study. These results demonstrate that both lytic (K8.1) and latent (LANA) KSHV transcripts are present at 3dpi in our infection system. This data is now included as Figure 3H in the revised manuscript. Additionally, we agree with the reviewer that validating that the lineages identified as susceptible to infection by flow cytometry should be confirmed by a method other than the GFP reporter. Thus, we have included lineage-specific real time PCR data in the revised manuscript. For all lineages except plasma cells, we sorted individual lineages at 3 days post-infection from three tonsils and performed nested RT-PCR for GAPDH, LANA and K8.1. This data is now included as Figure 3I in the revised manuscript and shows the presence of viral transcripts in all of the B cell lineages which show GFP positive cells in our flow cytometry analysis. For plasma cells, because of how rare they are in tonsil, we could not get enough material to do RT-PCR on a bulk sorted population so we used a single cell RT-PCR approach. This data is included in the revised manuscript as Figure 3J and shows the presence of KSHV transcripts in plasma cells. We have added text for these new data in both the results and discussion sections, as appropriate.

In response to the Reviewer’s final comment in this section we have performed analysis to examine the total number of each B cell lineage at baseline (pre-infection) and 3dpi in the Mock and KSHV-infected conditions to determine whether the culture system and/or KSHV infection is substantially altering the number of any particular B cell lineages. These results demonstrate that for most lineages KSHV infection does not substantially alter the overall level of the lineage compared to the matched Mock and baseline samples. The data show that our culture system does not favor the survival of CD138+ plasma cells, indicated by a significant decrease comparing baseline and Mock at 3dpi. However, this effect was lower in the KSHV-infected cultures. This result suggests that, rather than killing CD138+ cells or downregulating CD138 expression in the culture as the reviewer suggests, KSHV infection is either providing a survival advantage for CD138+ cells in the culture system or driving the differentiation of new CD138+ cells during infection. We were interested in examining whether this survival advantage is a result of direct infection of CD138+ cells or an indirect effect such as a paracrine effect. To examine this, we analyzed whether the CD138+ plasma cell survival effect (KSHV-Mock for the total population) was correlated with the frequency of infection of CD138+ cells in the same sample. This analysis showed no significant correlation between increased CD138+ cell numbers in infected cultures and the percentage of infection of the same subset in the culture. Thus, the data supports a conclusion of an indirect effect of KSHV infection on survival or differentiation of CD138+ cells in our tonsil lymphocyte cultures. These analyses are now included in the revised manuscript as Figure 2B and Figure 3C and are discussed appropriately in the results and discussion sections.

*Reviewer #2: Transmission of KSHV is presumed to be via saliva, but it is not clear what cells in the nasopharyngeal cavity are infected. This is a comprehensive analysis to determine which B cell subsets are permissive for KSHV infection in the tonsillar tissue of 40 donors of various ages. The authors utilize a GFP reporter virus coupled with high resolution multi-parameter flow cytometry to monitor cells that are infected over a 3 day co-culture experiment. Even though there is a wide range of GFP+ infection donors, several trends manifest. The major finding is that multiple subsets of B cells at different stages of differentiation are targets for KSHV- but of these, CD138+ plasma cells are infected at the highest frequency. Tropism was not accounted for merely based on syndecan/CD138 on the surface of plasma cells since soluble CD138 pre incubation with virions did not abrogate infection of the CD138+ cells. They also observed that a depletion of CD4+ T cells in donor samples with a higher baseline ratio of CD4 to CD8 cells led to a slight increase in GFP+ B cells. This paper further expands our understanding of KSHV tropism in primary B lymphocytes. However, there is no analysis of viral gene expression, cytokine profiles, or examination of T cell effector responses that might provide mechanistic insight. Critical technical controls are lacking to confirm that the virus adsorption with soluble CD138 and T cell ablation strategies were effective.*

We have responded to this reviewer’s more detailed comments expanding upon these concerns below in Part II.

*Reviewer #3: KSHV is a B cell tropic and lymphomagenic virus transmitted via saliva. Therefore, defining the specific B-cell tropism in the context of the tonsillar environment is essential to fully understand KSHV transmission and the pathobiology of de novo infection in the B-cell lineage with essential implications for KSHV induced lymphomagenesis. In this succinct and well-designed study, the Totonchy group uses a collection of 40 human tonsils to identify the B-cell lineage, which is most susceptible to KSHV infection. They found that CD138+ plasma cells, albeit constituting a small percentage of the B-cell lineage population are highly infectable by KSHV. They also provide data pointing to a role of CD138 in virus attachment and that T-cells present in the microenvironment may influence the outcome of infection. Thus, this is an important and significant study; mainly, since one of the markers of the PEL KSHV driven lymphomas is CD138, the study suggests that these lymphomas may arise upon infection of more mature and differentiated B-cells. It opens new avenues for the development of models of KSHV lymphomagenesis. A few experiments will make it even more robust and compelling. Particularly important would be characterizing the type of infection carried out by KSHV in the described population.*

The reviewer’s suggestion that we include data characterizing the type of infection is now included in Figure 3I&J. Please see our detailed response to Reviewer 1, above explaining these experiments. The results show LANA transcripts predominating in all lineages with only memory cells and transitional cells in 1 of 3 samples showing high K8.1 transcripts. Our single cell analysis shows that plasma cells are a mix of lytic and latent infection.

We apologize to the reviewer that, after we addressed all the excellent suggestions for additional data and analysis provided by this review, the study can no longer fairly be described as “succinct”.

**Part II – Major Issues: Key Experiments Required for Acceptance**

*Reviewer #1: 1. Infection determination: KSHV.219 expresses GFP off of the constitutively acvtive cellular (i.e. non-viral) EF-1α promoter. Thus, detection of GFP indicates only that the viral genome entered the cell nucleus but not that it established bona fide infection. To ascertain biologically relevant levels of infection (rather than simply entry, which is necessary but not sufficient for the establishment of infection), the authors would need to assess either latent and/or lytic native gene expression. For the latter, KSHV.219 would allow RFP to serve as a surrogate since it’s driven by the KSHV lytic PAN promoter. This point is important since it is absolutely conceivable that various tonsillar lymphocytes could be permissive to entry but not support downstream viral gene expression (and establishment of actual infection, whether latent or lytic).*

We agree with the reviewer’s critique and have included data in the revised manuscript to address the issue via examining viral gene expression in the bulk lymphocyte cultures (Figure 3H) and individual B cell lineages by RT-PCR (Figure 3I&J). These experiments and their results are described in more detail above in our response to Reviewer 1 and Reviewer 3 for Part I.

*2. Fig. 4: If the CD138 is going to be tested as a contributor to entry in this system, it would have been helpful to have a negative control, especially since no dose-response of the srCD138 is apparent. E.g. incubating with another recombinant surface protein; looking at the results from blocking CD138 on the surface of cells prior to addition of the virus; or showing that another virus not known to use CD138 is not blocked by the addition of srCD138. Overall, the authors conclude that the data are not particularly convincing so perhaps this fig. could be omitted altogether.*

We appreciate the concerns of the reviewer about the CD138 experiments. We believe that this data, although negative, is important given that Hahn et. al. showed CD138 can interact with KSHV gH in a non-lymphocyte system (reference 21 in the manuscript). We believe that our approach to these experiments represents the best approach available given that the specific biochemistry of any putative CD138 interaction with KSHV glycoproteins is unknown. We considered using a CD138 neutralizing antibody as the reviewer suggests, but the binding epitope of any given CD138 antibody may differ from the binding domain used by KSHV glycoproteins, in which case the antibody would not affect the interaction with KSHV glycoproteins and the assay would provide a false negative result. Using soluble recombinant protein does not have this issue because the majority of interaction domains that exist on the cell surface protein will be present in the soluble version, making the result more reliable given the limited information we have about the specifics of the putative interaction. Unfortunately, we know of no way to directly assess binding of srCD138 to virus particles. This was our rationale for using a dose-response approach. The reviewer is correct that the inconsistency of the inhibition and the lack of dose-response in these data support the conclusion that srCD138 does not bind to KSHV virions and thus CD138 is not used as an entry receptor for plasma cells. We have altered the results section (lines 275-280) to clarify our choice of method and our conclusions from this data.

*Reviewer #2: 1. Technical concerns with the infection experiments.*

*• A tonsillectomy is presumed to be in response to a medical issue. Were there any donors with specific conditions that led to disparate, skewed results as outliers? Were co-infections with adenovirus or EBV examined?*

As our tonsil specimens are de-identified medical waste material, we do not have access to the medical records of the tonsil donors. We agree that the influence of co-infections and commensal organisms may be a significant factor influencing the donor-dependent differences in susceptibility to KSHV infection. In fact, this analysis is the subject of a forthcoming study from our group where we will correlate bacterial and viral metagenomic data with KSHV susceptibility in our tonsil specimen cohort.

*• These cells were frozen prior to use and plasma cells have decreased recovery after freezing. Was the baseline flow analysis performed at the initial isolation prior to freezeback or subsequent to thawing after/concurrent with infection?*

The baseline flow cytometry analysis and the infections were all performed on material that had been freeze-thawed. Our limited access to tonsil specimens (3-4 specimens per month, on average) would make working exclusively with fresh cells unfeasible. Moreover, the large number of lymphocytes obtained from a human tonsil is sufficient for 5-20 individual experiments, depending upon the size of the tonsil, so using frozen cells is significantly less wasteful of the donor tissue. We believe that our exclusive use of freeze-thawed material in our analyses adequately controls for this factor, and even if plasma cells have a reduced recovery, we would argue that this effect would be consistent across samples, thus comparing the relative levels and susceptibility of plasma cells in different samples remains experimentally valid in our system.

*• As described for Figure 2A procedure, 1x10^6 naïve B cells were used for infection to normalize infection across donors. The authors should clarify whether uninfected naïve cells were added back to the co-culture to restore naïve B cell numbers or was there a constant ratio of infected naïve to bound cells across all donors?*

We would expect that in the KSHV infected conditions, the naïve B cells are a mixture of infected and uninfected cells at the time of co-culture. The ratio of naïve to bound cells would not be consistent across donors, but normalizing this would be impractical and would generally negate our effort in making the infections consistent across donors.

Given that the final co-cultures are 1x10^6 manipulated naïve plus 1x10^6 bound cells, one might expect that the co-cultures would over-represent naïve B cells compared to the unmanipulated baseline data. However, the new Figure 2B comparing overall frequencies of B cell lineages for baseline, Mock and KSHV infected conditions shows that levels of naïve B cells are consistently decreased in the Mock and KSHV conditions compared to baseline. This may reflect a proportion of naïve B cells failing to survive the infection protocol or differentiation of naïve B cells into another lineage in our culture system which are both consistent factors between Mock and KSHV conditions.

*• Are the naïve cells truly infected or are they just serving as carriers of cell-surface bound virions for the 3 day culture?*

We have no way of directly addressing this experimentally, but Figure 3B of the original manuscript shows that naïve B cells are not as susceptible to infection as other cell types in our co-cultures, which would support the conclusion that the naïve B cells are either primarily acting as carriers of cell-surface bound virions or are becoming infected and then differentiating in to a different lineage due to the culture system. Additionally the new RT-PCR analysis in Figure 3J indicates that Naïve cells are undergoing latent infection at 3dpi.

*• Do the bound cells have some baseline level of activation after the naïve B cell isolation? After incubation on the feeder cells?*

This is an excellent question, and we believe it is also addressed by our analysis included in the new Figure 2B comparing overall B cell frequencies in baseline, mock and KSHV-infected cultures. If the bound cells were activated during the isolation procedure, we would expect that there would consistently be increased frequencies of these lineages in the Mock condition compared to the baseline due to proliferation. Activation and proliferation of cells in response to the feeder cell system would be similarly reflected in increased numbers in the 3dpi conditions vs. the baseline. However, the data in the new figure show that this is not the case, and most lineages are not substantially altered in their frequencies at 3dpi compared to baseline. We discuss the issue of the culture system and activation of the cells extensively in our previous publication (Totonchy et al, 2018).

*• The shCD138 studies are not conclusive. How much recombinant CD138 was added? There was no verification that the CD138 bound to the virions. Can the CD138 on the targets cells be blocked?*

We discuss this issue above in reviewer #1’s point #2 in this section. The doses of CD138 are listed on the x-axis of the figure. Not knowing the specific binding domain for a putative interaction we believe the soluble recombinant protein is a better choice than a neutralizing antibody which may or may not have a binding site overlapping the site being used by KSHV glycoproteins. We also address the reviewer’s final point indirectly with the inclusion of heparinase treatment data in the revised manuscript Figure 4F-I. Heparinase treatment would remove CD138 from the cell surface and this treatment does not impair the infection of plasma cells in our system.

*• Figure 5. The increase in GFP+ cells is only increased by 20% and that is influenced heavily by 3 of the donors. Is this increase statistically significant in a paired T test for the CD4 depletion?*

Aggregating all 11 tonsil samples used for these experiments (as shown in Figure 6B of the revised manuscript), there is no statistically significant difference between non-depleted controls and either CD4 or CD8-depleted conditions. We stated in the results text in the original manuscript that the aggregated data was not statistically significant, but have added a statement to this effect with the statistical test in the figure legend. We commented on power analysis for the correlation data in the original figure legend, and concluded that for this dataset correlations of 0.7 and higher can be considered statistically significant. We have added a statement to this effect in the results section to clarify readers’ interpretation of these results.

*• Line 149: Why is T cell depletion data not shown? This is a critical control that should be included, minimally as a supplemental figure.*

At the reviewer’s request we have included a representative FACS plot showing the T cell depletions as Figure 6A in the revised manuscript. The other panels in this figure have been re-designated appropriately.

*2. Data analysis and presentation of the data*

*• Figure 1C- Is there a direct correlation between increased memory and loss of germinal center B cells in donors by age?*

The multiple correlation coefficient for Memory B cells and Germinal Center B cells by Age in our data set is 0.56, suggesting a moderate direct correlation between these two subsets with age.

*• Figure 2B is very difficult to interpret. The authors might place GFP on the y axis and break down the data as in Figure 1A by gender and race with a color code and then have a separate panel with GFP on y axis with the donor age bins on the x-axis as presented in Fig 1C. What statistical test was applied to determine a lack of correlation with age, gender, or race in Figure 2?*

We appreciate these comments from the reviewer and the opportunity to make our data presentation more clear. Our aim in Figure 2B was to show the frequency of samples in our cohort showing each level of infection, which is harder to visually interpret in the scatter graph in Figure 2C. We attempted to clarify this by replacing Figure 2B and 2C with a new Figure 2B showing a combination graph where the y-axis is shared between a scatter plot showing the relationship to age/sex/race on the left and the frequencies at each level of infection as a histogram on the right. We have added more information to the results text for this figure (lines 130-134) specifying the statistical tests employed to demonstrate a lack of correlation between the donor demographic information and susceptibility to KSHV infection.

*• Figure 3 highlights the CD138 as having the highest percentage of infection, but this does definitively demonstrate that CD138+ cells are the major latency reservoir. An analysis based on total numbers in the tonsil tissue (% infected * frequency of total) provided as a table or additional graph is needed to describe how the infected cell subsets contribute to the overall viral load in the tonsil tissue.*

In our manuscript, we do not make the claim that CD138+ cells are a major latency reservoir, only that they represent a highly targeted population based upon the percentage of infection within the subset. We did not include the frequency of individual B cell lineages within the GFP+ population in the original manuscript because between samples comparisons of this data are confounded by the variable frequency of each subset in each specimen (as shown in Figure 1B). However, the reviewer makes an excellent suggestion here to calculate the within-sample frequency of total as a valid way of summarizing the distribution of GFP among rather than within subsets. We have included data calculated in this manner as new panels Figure 3D&E in the revised manuscript and have included text in the results section as appropriate. This analysis shows that the distribution of subsets within the GFP+ population is variable between donors, and is not heavily influenced by overall susceptibility (Figure 3D, order of samples from highest to lowest susceptibility). The data further show that germinal center, transitional and memory cells make the largest contributions to overall infection. Plasma cells and MZ-like cells are intermediate contributors, while double negative, naïve and plasmablast make up a minor proportion of the infected cells.

*• This group previously reported an Ig lambda light chain bias in the KSHV+ cells and an increase in IL-6 production in the infected cultures. There are likely other features of B cell maturation, differentiation and survival that are influenced by infection. Did the authors observe any bias in Ig light chains of the plasma cells in this study?*

We sympathize with the reviewer’s curiosity about the Ig light chains in the current study. However, the number of cell surface antibodies needed to identify all of the B cell lineages in this study did not allow us to include additional stains for light chains given the flow cytometry instrumentation at our disposal. We hope to expand our analysis in the future to include both light chains and immunoglobulin isotypes as well as finer distinctions within subsets that were not feasible in this analysis.

*• Figure 3C examines how the percent GFP in a given subset correlates with baseline frequency, but a similar analysis with GFP correlation to 3 dpi would also be helpful and complement Fig 3A and 3B. Does the negative 0.4 correlation of plasma cells with memory in Fig 3C indicate the virus is shifting memory to plasma in the culture or that if there are more memory at baseline there will just be fewer plasma cells to infect? Does the subset distribution (e.g. CD27) change between 0 and 3 dpi with KSHV infection? Do marginal zone or plasma cells increase?*

Based on the reviewer’s suggestion, we have included an additional correlation plot similar to Figure 3C (now Figure 3F) as new panel Figure 3G in the revised manuscript correlating within-subset infection frequency with the frequency of each subset at 3dpi in the KSHV-infected condition, and have included a description of the results in the manuscript, as appropriate. We believe that the remaining questions posed by the reviewer in this point are addressed by the inclusion of the new Figure 2B comparing overall B cell frequencies in baseline, mock and KSHV-infected cultures, which shows that there is very little difference between the baseline frequencies of subsets and their frequencies at 3dpi with the exception of naïve and transitional lineages. There is an increase in plasma cells in KSHV compared to Mock at 3dpi which could indicate either a survival advantage of infection or, as the reviewer suggests, differentiation of another subset into CD138+ cells in response to infection. There is not an obvious decrease in any particular lineage seen in Figure 2B or negative correlation in Figure 3G which would clearly indicate the latter scenario. Experiments specifically addressing the issue of trans-differentiation of infected populations in our mixed lymphocyte culture system are currently underway and will be the subject of a forthcoming study.

*• Figure 4. The % changes in GFP are variable by donor. Is this attributed to a very low number of cells being infected such that any small changes can lead to positive or negative changes in the % infected which are not biologically meaningful? Were the 0 dose samples infected with a mock virion stock pre-incubated with the rhCD138 vehicle? Could the individual donors be color-coded across the subsets of the figure?*

We agree with the reviewer’s interpretation of these results. Small changes in GFP positivity are a direct result of the relatively low percentage of infection at this timepoint. As discussed above, we have modified the text in the results section (lines 306-308) to clarify that we believe the inconsistency of these results from sample-to-sample as well as the overall lack of a dose-response profile indicates that CD138 is not an attachment factor for infection of plasma cells. Moreover, we have included additional data supporting this conclusion in Figure 4F-I showing that removal of HSPG prior to infection by heparinase treatment does not affect infection of plasma cells or other B lymphocyte lineages. The zero dose samples in the CD138 experiments were infected with KSHV virions that were mock treated (i.e. had been handled and incubated the same as the virions that were pre-incubated with doses of srCD138). As the reviewer requested, we have revised Figure 4 to include color coding for the individual tonsil samples.

*• The T cell composition of supplemental figure 2 was not well-incorporated into the paper. Did any particular T cell subsets correlate with % infected B cells?*

We agree with the reviewer’s critique and agree that more can be done to incorporate the T cell data into the discussion of KSHV tropism and the mechanisms for its donor-to-donor variability. In response to this comment, we have moved the data from Supplemental Figure 2 into the main body of the paper. Panels A and B have been included in Figure 1 as panels D and E and panel C of the original supplemental figure is now Figure 5A – making the original Figure 5 now Figure 6 and included additional panels showing pairwise correlations between KSHV infection of B lymphocyte linages and baseline frequencies of T cell subsets (Fig 5B), as well as scatter plot data for highly significant (r ≥ 0.5) correlations between CD4+ T cell subsets and infection of CD138+ plasma cells (Fig 5C). These data are now also discussed in the results and discussion sections. They show no significant correlations between baseline levels of T cell subsets and overall susceptibility to infection with KSHV. However, there are significant correlations to the targeting of particular cell types by KSHV infection suggesting that the T cell microenvironment may not influence overall susceptibility but does influence the distribution of KSHV infection among cell types, which could theoretically affect the establishment of infection *in vivo* depending on the relative contribution of different lineages to that process*.* Moreover, this data is consistent with our data in Figure 6 (originally Figure 5) showing a more profound effect of CD4 T cell depletion on infection of plasma cell subsets.

*• With regard to mechanism, were viral genes profiled to examine differences in the major subsets (naïve, marginal zone, germinal center, memory plasma cells? Did any cytokine profiles correlate with B cell subsets, T cell subsets (before/after depletion), or percent infections?*

We have now included gene expression profiles in Figure 3H-J of the revised manuscript, as suggested by other reviewers and described above in our response to reviewers #1 and 3 in Part I. We have done some work on cytokine secretion in our system, but believe that this is outside the scope of the current manuscript. This issue is the subject of a forthcoming manuscript which will satisfy the reviewer’s curiosity on this subject and provide enough space for us to include mechanistic insight into the important question of cytokines and their influence on the establishment of KSHV infection.

*3. Interpretations and incorporation of findings into larger field*

*• The results section is quite brief and does not fully explain the data or develop a meaningful narrative.*

We have endeavored to strengthen the results section as the reviewer suggests.

*• What stage of virus infection does the GFP reporter gene indicate? If an artificial promoter, could the signal strength differ with cell type and activation status?*

As pointed out by Reviewer #1 and discussed extensively above, the GFP expression is driven by EF1-alpha promoter in the viral genome. Evidence in the literature and our own observations suggest that cells undergoing lytic replication do have higher levels of GFP expression due to more copies of GFP present on the replicating viral genomes, but given that others have shown variable silencing of GFP reporters in recombinant KSHV in primary cells we believe that GFP levels based on signal intensity by flow cytometry should not be over-interpreted.

*• Please expand on the discussion comment that these observations complement the finding of Myoung et al. showing that CD4+ T cells control lytic reactivation of KSHV in primary B cells.*

We have clarified this comment in the discussion.

*• The reader needs more information on the UPR and KSHV discussion point; it is rather vague without referring to the cited paper.*

We have expanded this part of the discussion section, as the reviewer suggested.

*• How does this subset distribution relate the EBV in cell culture or MHV68 in vivo?*

To our knowledge, the subset distribution of EBV has not been studied using extensive immunophenotyping to identify specific lineages as we have done here. Moreover, comparisons to MHV68 infection would be relatively artificial given that it initially establishes infection in B cells in the spleen or lymph nodes of mice as opposed to the oral lymphoid tissues. Nonetheless, we have included text in the discussion section of the revised manuscript to address both of these points.

*• How would CD20+ plasma vs CD20- plasma vs plasmablasts contribute to infection/transmission and pathogenesis/disease?*

We have included some thoughts on this interesting question in the discussion section of the revised manuscript (lines 438-445)

*Reviewer #3: 1. An essential aspect of completing this study is the characterization of the type of KSHV infection established in the CD138+ Plasma cells. I think that since the researchers are using a r219KSHV, it is a missed opportunity that they do not try to procure a proper cytofluorometer to take advantage of this opportunity. If that continues not to be affordable, they can at least use qRT-PCR of a panel of KSHV RTA and early lytic, late lytic and latent genes and by test virion production by testing the infectivity of the supernatants in 293 cells.*

We have included lineage-specific single cell RT-PCR in Figure 3I&J of the revised manuscript to address this important point that was also made by Reviewer #1 and is discussed extensively above.

*2. This group has previously shown the ability of KSHV infection to manipulate the Ig recombination machinery in naïve B cells and other groups had shown the potential of KSHV infection to induce Ig heavy chain class switch recombination. Therefore it will be interesting to determine the existence of changes in the Ig class population composition upon infection of CD138+ plasma cells.*

We agree that this analysis would be interesting. However, we believe that this is outside the scope of the current study and technically difficult due to accomplish. To truly demonstrate BCR revision as shown in our previous paper, infection must be done in the context of pure IgK population and the fact that plasma cell numbers are already low in our system would confound the analysis if we tried to subset them by light chain class. Thus, this analysis would also need to be done by a single cell method. As we state above, we hope to expand our analysis in the future to include both light chains and immunoglobulin isotypes as well as finer distinctions within subsets that were not feasible in this analysis once we have access to more sophisticated instrumentation.

*3. The results with the srCD138 showing the potential involvement of this receptor in KSHV attachment are interesting. It is worthy to notice that since this mechanism is mediated via HSPGs the researchers may not obtain the kind of clear-cut results obtained when testing a major internalizing receptor since HSPGs generally contribute to binding to the cell surface so that the specific receptor can internalize the virus. It will be interesting to test whether heparin treatment of the virus or heparitinase/ heparanase pre-treatment of the target cells mimics the results obtained with the soluble CD138.*

This is an excellent suggestion by the reviewer to include heparinase treatment to complement and validate the srCD138 experiments. We have performed these experiments in both tonsil lymphocytes and fibroblasts, and have included the data in Figure 4D-4I of the revised manuscript. As expected, heparinase treatment decreased KSHV infection in fibroblasts. However, consistent with our srCD138 data, heparinase treatment of lymphocytes does not consistently lower the rate of infection in our system. We were concerned about the detection of plasma cells after heparinase treatment given that the CD138 marker we use to identify them by flow cytometry would also be removed by the treatment. However, by three days post-infection heparinase treated cultures had similar levels of plasma cells detected compared to non-treated controls, demonstrating that CD138 expression is restored on plasma cells prior to our analysis. The 3dpi analysis shows that heparinase treatment does not consistently decrease the proportion of plasma cells infected by KSHV in our system, further indicating that the CD138 HSPG is not required for infection of plasma cells and providing the finding that KSHV does not generally use HSPG as an attachment factor for infection of B cells which is consistent with the current literature from lymphoma cell lines. We have added text and references to the discussion section to expand upon this point (lines 456-459)

**Part III – Minor Issues: Editorial and Data Presentation Modifications**

*Reviewer #1: Fig. S1A. B and T cells based on 31 and 32? To what do 31 and 32 refer?*

We have altered the figure legend text to clarify that the B cell and T cell gating schemes were adapted from references 31 and 32.

*‘fig. 2 B. It is unclear in the text and legend what each filled-in circle represents. If it indicates a separate tonsil, then why are there >40 circles? Suggest clarification in legend.*

We have clarified this point in the figure legend text as the reviewer suggested. N=50 from 40 tonsil specimens with biological replicates for 10 specimens.

*Line 97: susceptibility of “naïve” (insert “naïve”) B cells to KSHV…*

What is represented in this graph is the total infection of all viable, CD19+ B cells at 3dpi. This value includes many lineages other than naïve B cells, thus the addition of naïve to this sentence would not be an accurate description of the data in the figure.

*Lines 118-121*

*Conclusion (1) and (2) is consistent with their data but also consistent with findings from previous work from other labs. Consider inserting such a statement.*

We agree with the reviewer that Conclusion 1 is not novel in the literature and have included a statement to that effect in the text here. It should be noted that we address this point in more detail in the Discussion section. We are not aware of a previous study that has characterized donor-dependent differences in overall susceptibility of tonsil lymphocytes to KSHV infection, so we have not added anything to this point.

*Line 189. Not all studies used stimulation of target cells in in vitro infection systems.*

The statement in the original manuscript reads “With the exception of Bekerman et. al, all of the above-mentioned studies employed some kind of activating agent (PHA stimulation or CD40L stimulation) to manipulate the activation and/or proliferation of cells *in vitro”.* We believe this statement is accurate. The studies referenced in the preceding paragraph all used some form of stimulation prior to or after infection except for the Bekerman et. al. study.

*Mx discussion might be improved if authors integrated their data with earlier reports of tonsilar KSHV+ cells demonstrating plasmablast-like phenotype.*

This is an excellent suggestion. We have added text to the discussion addressing this point (lines 391-396).

*Reviewer #2:*

*• Line 45: ‘unstimlated’*

This error has been corrected.

*• Referred to as srCD138 in text, but rhCD138 in figure 4.*

This error has been corrected. This reagent is now referred to as srCD138 throughout the manuscript.

*• The frequent use of the singular ‘tonsil’ seems awkward.*

We have reviewed our wording throughout the manuscript and have revised the stand-alone references to “tonsil” to “tonsils”, but have left “tonsil-derived”, “the tonsil”, “tonsil specimens”, “tonsil tissue” and “tonsil lymphocyte(s)” as singular.

*• Figure legends are a bit sparse and do not enable the figures to stand alone.*

We have endeavored to add detail to the figure legends in response to this comment.

*• Figure 5 y axis legend labels: specify percent of non-depleted control*

This error has been corrected.

*• Figure 5A: specify what the colors indicate.*

This is specified in the figure legend for Figure 5A “colors indicate individual tonsil specimens (n=11)”

*• No label 7 in supp figure 1*

This error has been corrected.

*• Hard to distinguish labels with specific boxes in supplemental figure*

In response to this comment, we have added color to the gate boundaries and corresponding numbers to help clarify the lineage definitions on the plots.

*Reviewer #3: Since this is a succinct study, it will help the reader that the supplementary data are presented in the main text figures. These include patient data in classic table form, B-cell lineages etc.*

In response to this suggestion, we have added tables to the main text including donor data in table form (Table 1 in the revised manuscript) and a table of B and T cell lineage definitions (Table 2 in the revised manuscript). The FACS plots detailing lineage definitions remain in Supplemental Figure 1A because we believe that a smaller subset of PLoS Pathogens readers will be interested in examining this level of detail for the FACS analysis. We have moved the data in Supplemental Figure 2 to the main text as Figure 5, and Figure 5 of the original manuscript is now Figure 6.
